# Bioinformatics-Driven, Plant-Based Antibiotic Research Against Quorum Sensing and Biofilm Formation in *Pseudomonas aeruginosa* and *Escherichia coli* Multiresistant Microbes

**DOI:** 10.3390/biom16020197

**Published:** 2026-01-27

**Authors:** Serena Rosignoli, Elisa Lustrino, Olga Shevchuk, Serena Rinaldo, Elisabetta Rubini, Alessandro Paiardini, Ivana Carev

**Affiliations:** 1 Centre for Regenerative Medicine “Stefano Ferrari”, Department of Life Sciences, University of Modena and Reggio Emilia, 41125 Modena, Italy; serena.rosignoli@unimore.it; 2 Department of Biochemical Sciences “A. Rossi Fanelli”, University Sapienza, P. le Aldo Moro 5, 00185 Rome, Italy; elisa.lustrino@uniroma1.it (E.L.); serena.rinaldo@uniroma1.it (S.R.); elisabetta.rubini@uniroma1.it (E.R.); 3 Group of Immunoproteomics, Department of Immunodynamics, Institute for Experimental Immunology and Imaging, University Duisburg-Essen, 45141 Essen, Germany; olga.shevchuk@uk-essen.de; 4 Faculty of Chemistry and Technology, University of Split, 21000 Split, Croatia

**Keywords:** antibiotics, antimicrobial resistance, bioinformatics, *Escherichia coli*, UPEC, *Pseudomonas aeruginosa*, quorum sensing (QS), biofilm formation

## Abstract

Quorum-sensing (QS) systems play a crucial role in regulating virulence, biofilm formation, and antibiotic resistance in clinically relevant microbes. This review explores the potential of QS systems as targets for developing novel plant-based therapeutic strategies using bioinformatics, aimed at combating highly pathogenic bacteria: uropathogenic *Escherichia coli* (UPEC) and *Pseudomonas aeruginosa*. We examine the key components and molecular pathways of QS systems in these microbes, including autoinducer synthases, receptors, and regulatory proteins. In UPEC, we discuss the LuxS-dependent autoinducer (AI)-2 system, while for *P. aeruginosa*, we analyze the more complex interconnected Las, Rhl, and PQS circuits. We highlight how these systems control the expression of virulence factors and contribute to biofilm formation, emphasizing their importance in pathogenesis. Furthermore, we explore bioinformatics approaches for identifying and characterizing QS components, i.e., by predicting protein structures and interactions. The potential of in silico screening for QS inhibitors is also discussed, along with challenges and opportunities in targeting QS systems for therapeutic interventions. By integrating microbiological, molecular, and computational perspectives, this review aims to provide insights into the application of bioinformatics in understanding and targeting QS systems in these clinically significant pathogens. The goal is to facilitate the development of novel anti-virulence approaches in search of novel antibiotics that could complement or replace traditional antibiotic treatments, addressing the growing concern of antimicrobial resistance in these clinically relevant microbes.

## 1. Introduction

Antimicrobial resistance (AMR) represents one of the most pressing global health challenges of our time, threatening the very foundations of modern medicine. The increasing prevalence of drug-resistant pathogens not only jeopardizes our ability to treat common infections but also compromises critical medical procedures that rely on effective antimicrobial prophylaxis. This crisis extends far beyond human health, impacting animal welfare, agricultural productivity, and food security, with significant economic loss including escalating healthcare costs, reduced workforce productivity, and diminished agricultural output. The World Health Organization (WHO) and European Commission (EC) have identified AMR as a major global health threat, projecting that without intervention, annual global deaths attributed to AMR could reach a staggering 10 million by 2050. In the European Union (EU) alone, AMR is responsible for over 35,000 deaths annually, placing immense strain on healthcare systems and dramatically increasing morbidity and mortality rates associated with previously treatable infections [1,2,3,4].

**Figure 1:**
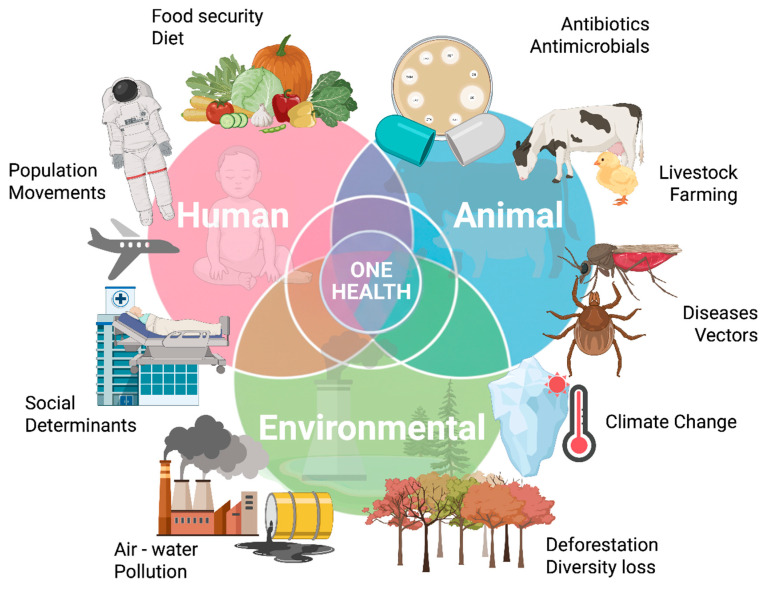
The One Health concept and its interconnected domains. The One Health framework illustrates the interdependence of human, animal, and environmental health. Human health (pink) is influenced by factors such as diet, food security, antibiotic and antimicrobial use, social determinants, and population movements. Animal health (blue) is affected by intensive livestock farming, disease vectors, and antimicrobial usage. Environmental health (green) encompasses key elements including climate change, air and water pollution, deforestation, and biodiversity loss. The overlapping areas highlight the complex interactions among these domains, emphasizing the need for integrated, multidisciplinary approaches to address global health challenges. Illustrations adapted from [7].

However, this crisis also presents significant opportunities for innovation and progress. The urgent need for novel antimicrobials has spurred increased focus on research and development, guided by the WHO’s priority list of pathogens. The European Commission’s recognition of AMR as a top priority has opened avenues for increased resources and investments in antimicrobial innovation. Additionally, the adoption of a “One Health” approach provides a comprehensive framework for addressing AMR across human, animal, and environmental sectors (Figure 1). This challenging landscape not only demands the development of new antimicrobials but also encourages the exploration of safe, effective alternatives to conventional treatments, potentially revolutionizing our approach to AMR [5,6].

The urgency of developing such alternative strategies stems from the growing ineffectiveness of traditional antibiotics. The World Health Organization’s priority list for antibiotic research and development highlights six major pathogens—*Enterococcus faecium* (*E. faecium*), *Staphylococcus aureus* (*S. aureus*), *Klebsiella pneumoniae* (*K. pneumoniae*), *Streptococcus pneumoniae* (*S. pneumoniae*), *Acinetobacter baumannii* (*A. baumannii*), *Pseudomonas aeruginosa* (*P. aeruginosa*), *Enterobacter* spp. and *Escherichia coli* (*E. coli*) (collectively known as the ESKAPE-E pathogens), which are responsible for a large share of AMR-associated deaths worldwide. These pathogens have evolved multiple resistance mechanisms that render many conventional antibiotics increasingly ineffective, driving the search for innovative therapeutic solutions [8,9,10].

In this search, plants represent an extraordinary but still largely untapped source of new antimicrobial agents. Over millions of years of evolution, plants have developed a remarkable diversity of bioactive molecules to defend themselves against microbial attacks [11,12]. These natural products, often characterized by unique chemical structures and multitarget activity, hold great promise for the discovery of compounds capable of interfering with quorum sensing and biofilm formation. Exploring this potential requires not only classical pharmacognostic approaches but also the integration of modern computational and experimental techniques. Here, bioinformatics plays a pivotal role. Machine learning algorithms, combined with specialized bioinformatics databases, can predict protein–ligand interactions and identify potential inhibitors active against resistant strains [13,14]. In parallel, advances in phytochemistry, microbiology, biochemistry, and proteomics provide powerful laboratory tools for the rapid screening, isolation, characterization, and experimental validation of plant-derived compounds targeting biofilms and QS systems.

Ultimately, the real innovation lies in bringing these computational and experimental methodologies together into a single, integrated workflow. Such a multidisciplinary, systematic, and sustainable approach could dramatically accelerate antimicrobial drug discovery, reducing the current development timeline of over 12 years and costs approaching 2.6 billion dollars, while paving the way toward novel, effective, and eco-sustainable therapies in the fight against AMR.

This report summarizes the approaches used in the past decade to discover and characterize plant-derived anti-QS and anti-biofilm agents, from ethnobotanical selection of medicinal plants to laboratory extraction, assay techniques, and identification of active compounds. Key phytochemical classes (flavonoids, alkaloids, terpenoids, phenolics, etc.) and recent examples from peer-reviewed studies (2015–2025) are highlighted to illustrate the progress in this field. This review also aligns with a broader research effort aimed at identifying and evaluating innovative strategies to combat AMR pathogens through the discovery of new drugs. Central questions guiding this inquiry include: Which new compounds or drug combinations show the most promise for managing AMR infections? What interdisciplinary approaches, combining bioinformatics, microbiology, phytochemistry and proteomics, can be adopted to enhance the efficacy and precision of therapeutic interventions?

## 2. Biofilm and Quorum Sensing in Antibiotic Resistance

Central to the challenge of AMR is the role of bacterial biofilms, which significantly contribute to the persistence of infections. Biofilms provide a protective extracellular matrix that impedes antibiotic penetration and fosters metabolic heterogeneity, thereby promoting antibiotic tolerance [15,16,17]. This structure of sessile communities of microorganisms, embedded in a self-produced extracellular polymeric substance (EPS), provides a protective environment that enhances resistance to antimicrobial agents and is implicated in about 80% of microbial infections in the human body. Biofilm-mediated resistance represents therefore one of the greatest challenges in the treatment of bacterial infections, particularly urinary tract infections (UTIs), which are among the most frequent bacterial infections worldwide. Within this context, UPEC and *P. aeruginosa* are responsible for between 50% and 90% of cases, depending on the population and type of infection. Their ability to form biofilms allows them to persist within the urinary tract, evade host immune responses, and resist conventional antibiotic therapies. Addressing this biofilm-mediated resistance is therefore a crucial step in the global effort to combat AMR [18,19,20,21].

Quantitative analyses reveal that minimum biofilm eradication concentrations (MBECs) are significantly higher than planktonic MICs, highlighting the biofilm-specific resistance phenotype [22]. Additionally, biofilm formation is frequently associated with virulence factors and genetic determinants such as fimbrial adhesion genes, underscoring its multifaceted role in pathogenesis [23]. However, many studies rely on in vitro biofilm models that may not fully replicate the complex in vivo urinary tract environment, limiting physiological relevance. Furthermore, heterogeneity in biofilm quantification methods and classification systems complicates cross-study comparisons [24].

A key mechanism underlying biofilm formation and maintenance is QS, a sophisticated bacterial communication process that enables populations to coordinate gene expression according to cell density. Through the production, release, and detection of small signaling molecules known as autoinducers, bacteria collectively regulate behaviors such as virulence factor expression and biofilm maturation.

This group behaviour was first described in *Vibrio fischeri* (*V. fisheri*), as a strategy to regulate the bioluminescence, luciferase-mediated phenomenon [25]. The luciferase operon is positively regulated by the LuxR transcription factor in response to the *N*-acyl-homoserine-lactone (AHL) signal molecule. AHL was defined as an autoinducer since the bacterium itself produces this molecule through the synthase activity of the LuxI enzyme; the autoinducer is released in the extracellular space and accumulates in a cell-density-dependent manner. Once it reaches a threshold level (“quorum”), the autoinducer re-enters the cell via a dedicated transporter and binds to its intracellular receptor LuxR. The autoinducer-bound form of LuxR is competent to positively control the target promoters [26,27].

**Figure 2:**
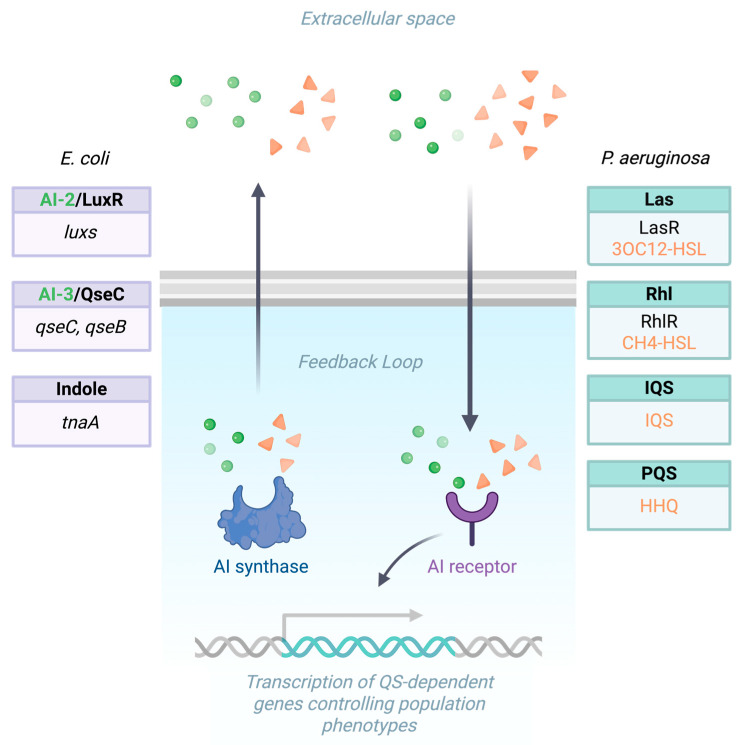
Quorum-sensing (QS) systems regulating biofilm formation in *E. coli* and *P. aeruginosa.* Schematic representation of the main quorum-sensing circuits in two clinically relevant pathogens. In *E. coli* (left, blue), three QS systems coordinate biofilm formation and virulence regulation: (i) the AI-2/LuxS system, involving the luxS gene and the Autoinducer-2 (AI-2) signal molecule; (ii) the AI-3/QseC system, mediated by qseC and qseB genes and the AI-3 signal; and (iii) the indole signaling system, based on tnaA and the indole molecule, which modulates biofilm formation and antibiotic resistance. In *P. aeruginosa* (right, green), four interconnected QS systems—Las, Rhl, PQS, and IQS—form a hierarchical regulatory network controlling biofilm development and virulence. The Las system (LasR/3OC12-HSL) activates downstream Rhl (RhlR/C4-HSL) and PQS (HHQ) systems, while IQS ensures QS activity under stress or nutrient-limited conditions. Together, these systems illustrate how bacterial communication networks orchestrate cooperative behaviors essential for infection and persistence.

A summary of the rationale of the peculiar signal transduction is reported in Figure 2.

Because QS directly influences biofilm development, disrupting these signaling pathways represents a promising strategy to prevent or weaken biofilm formation, offering an alternative route to control infections without imposing the selective pressure that accelerates resistance [28,29,30,31]. Disrupting these systems can attenuate pathogenic behavior, without necessarily killing the bacteria, thereby reducing selective pressure for resistance.

In clinically relevant pathogens such as UPEC and *P. aeruginosa*, QS plays a central role in regulating processes critical for infection and survival in the host. For example, *E. coli* employs three main QS systems that play crucial roles in biofilm formation and virulence regulation. The AI-2/LuxS system, involving the luxS gene and the autoinducer-2 (AI-2) signal molecule, regulates biofilm formation and virulence factor production. The AI-3/QseC system, with key genes qseC and qseB and the not fully characterized AI-3 signal molecule, is involved in biofilm formation and virulence regulation. Lastly, the Indole signaling system, centered around the tnaA gene (encoding tryptophanase) and using indole as the signal molecule, modulates biofilm formation and antibiotic resistance [32,33] (Figure 2). *P. aeruginosa*, in turn, employs four interconnected quorum-sensing systems that regulate biofilm formation and virulence: the Las, Rhl, PQS, and IQS systems. The Las system, considered the primary or master regulator, is based on the transcriptional activator LasR and its autoinducer *N*-3-oxododecanoyl-homoserine lactone (3OC12-HSL). It activates the expression of multiple virulence genes, including those coding for elastase, proteases, and exotoxins, and also initiates the hierarchical activation of the other QS circuits [34]. The Rhl system, centered on the regulator RhlR and its signaling molecule *N*-butanoyl-homoserine lactone (C4-HSL), functions downstream of Las and modulates rhamnolipid synthesis, swarming motility, and secondary metabolite production. The PQS system (Pseudomonas Quinolone Signal), mediated by the signal molecule 2-heptyl-3-hydroxy-4-quinolone (PQS) and its precursor HHQ, integrates with both Las and Rhl systems to fine-tune virulence gene expression, iron acquisition, and oxidative stress responses [35]. Finally, the IQS system (Integrated Quorum-Sensing signal), a more recently identified circuit, allows *P. aeruginosa* to maintain QS-regulated functions even under phosphate-limited conditions or when the Las system is impaired, thus ensuring robust control of virulence and persistence within hostile environments such as biofilms (Figure 2) [36]. Together, these four systems form a highly adaptable and resilient regulatory network that enables *P. aeruginosa* to survive in diverse ecological niches and persist in chronic infections, making it one of the most challenging pathogens in clinical settings [37].

As mentioned above, these two bacteria are relevant to multidrug-resistant infections; moreover, they represent model systems for studying the biochemistry of QS and biofilm formation, as acknowledged by the literature. A detailed description of these systems, with their role in biofilm maintenance, antibiotic tolerance and resistance, is therefore reported below.

## 3. *Pseudomonas aeruginosa*

### 3.1. Group Behavior in P. aeruginosa: Tolerance and Resistance to Antibiotics

*P. aeruginosa* represents the model organism for studying quorum sensing (QS) and biofilm-mediated infections. Among ESKAPE pathogens, it is a critical multidrug-resistant bacterium responsible for severe infections, particularly in nosocomial and immunocompromised patients, and a major cause of death in cystic fibrosis [38]. In the urinary tract, *P. aeruginosa* can establish chronic infections through its ability to adhere to epithelial cells, colonize catheters and urinary devices, and form robust biofilms that are difficult to eradicate [39].

Within biofilms, adverse conditions such as antibiotic exposure and nutrient limitation promote the selection of tolerant population and/or persister cells, a dormant, metabolically inactive subpopulation that survives antibiotic treatment without classic genetic resistance [40,41,42]. Persisters contribute to chronic and recurrent UTIs by re-establishing infection after therapy cessation [43]. Unlike resistance, which involves genetic modifications such as de novo mutations or horizontal gene transfer, tolerance allows the population to survive at high antibiotic concentrations without increasing the minimum inhibitory concentration (MIC) [44]. This tolerance is particularly evident in the deeper biofilm layers, where nutrient and oxygen gradients shape bacterial phenotypes, making tolerance the dominant cause of antibiotic failure in biofilm-associated infections [45,46,47,48,49].

Importantly, QS has been shown to promote antibiotic tolerance in *P. aeruginosa* biofilms following exposure to tobramycin and meropenem [50,51,52], in part by inducing DNA release via autolysis to strengthen the biofilm matrix [53]. During chronic infections, QS thus drives a transition from moderate drug tolerance to stable multidrug resistance [54]. Resistance mechanisms include extended-spectrum β-lactamases (ESBLs), carbapenemases, and aminoglycoside resistance mediated by 16S rRNA methylases [55,56].

Beyond acquired resistance, *P. aeruginosa* relies on intrinsic defenses (efflux pumps and outer membrane porins), all regulated by the QS network [57,58,59,60,61,62,63,64,65]. Efflux systems such as mexAB-oprM, mexCD-oprJ, mexEF-oprN, mexGHI-opmD, and PA1874-1877 contribute not only to antibiotic extrusion but also to the export of QS molecules and biofilm components. Additionally, QS controls outer membrane vesicle production, which facilitates antibiotic resistance, virulence, and intercellular communication [66,67,68,69,70].

QS also orchestrates the production of virulence factors that exacerbate urinary tissue damage. These include hydrolytic enzymes (elastase, proteases), rhamnolipids that disrupt epithelial tight junctions, and redox-active molecules such as phenazines and cyanide that induce oxidative stress in host cells [34,71,72,73,74,75]. Furthermore, the type III secretion system (ExoS, ExoT, ExoU, ExoY) and exotoxin A, all QS-controlled, directly target host defense mechanisms and promote tissue invasion [76,77,78,79].

Collectively, these features highlight how *P. aeruginosa* successfully exploits its QS network to integrate biofilm formation, antibiotic tolerance, resistance, and virulence—establishing a persistent phenotype to finally adapt and survive in multiple infection sites, despite the antimicrobial treatment [50,80].

### 3.2. QS Systems in P. aeruginosa

*P. aeruginosa* QS network includes four key systems: Las, Rhl (both based on acyl-homoserine lactones), pqs (based on quinolone signals), and IQS. As mentioned above, these systems regulate the expression of numerous genes to orchestrate many phenotypes in the population, including virulence and tolerance, making *P. aeruginosa* a model organism for studying bacterial social behavior. The main autoinducers synthesized by *P. aeruginosa* are *N*-(3-oxo-dodecanoyl)-L-homoserine lactone (3-oxo-C12-HSL, OdDHL), *N*-butanoyl-L-homoserine lactone (BHL or C4-HSL), 2-heptyl-3-hydroxy-4-quinolone (PQS, Pseudomonas quinolone signal), together with its precursor 2-heptyl-4-hydroxyquinoline (HHQ) and 2-(2-hydroxyphenyl)thiazole-4-carbaldehyde. These systems build an interconnected network of concerted regulation of gene expression, with the LasI/LasR found at the top of a hierarchical circuit, to finely tune the cell phenotype and finally adapt to a plethora of environmental conditions [81]. Las and Rhl systems are homologous to LuxI/LuxR, including a LuxI-type synthase and a LuxR-type receptor, and both systems respond to increased cell-density within a certain community [82].

The transcriptional regulator LasR in complex with its cognate autoinducer 3-oxo-C12-HSL induces (i) the expression of virulence factors such as protease and elastase (encoded by *lasA* and *lasB*, respectively), alkaline protease, lectins (encoded by *lec A* and *lecB*), pyocyanin either alone or in coordination with other branches of QS, and (ii) the expression of the *rhl*, *pqs* and *iqs* systems (in a cell density dependent manner). It should be mentioned that this hierarchy could be bypassed under specific conditions, such as phosphate limitation [83].

The RhlI/RhlR system, in response to C4-HSL, contributes to the production of the aforementioned virulence factors, thus de facto corroborating the Las response, and of rhamnolipid (including activation of rhlAB encoding a rhamnosyltransferase) and hydrogen cyanide [84]. Moreover, C4-HSL-RhlR complex sustains a second positive feedback loop by controlling RhlI expression. RhlI-independent regulons are controlled by RhlR, including biofilm formation and virulence factor genes [85]. There is a third *P. aeruginosa* regulator responding to acyl-homoserine-lactones named QscR, whose role is to dampen or delay the activation of LasR and RhlR-controlled genes [86]. Recent data indicate that QscR regulates a single operon, to finally raise the QS threshold of the population as a sort of “brake” on QS autoinduction [87]. Interestingly, QscR shows a broad signal specificity, including lactones from other species, allowing *P. aeruginosa* to tune its own QS readout in a multispecies environment [88].

Downstream of Las/Rhl systems, the Pqs system responds to 4-hydroxy-2-alkylquinolines (HAQs), including the aforementioned HHQ and PQS. These are synthesized by enzymes encoded in the pqsABCDE and phnAB operons, with the enzyme PqsH converting HHQ into PQS. Both HHQ and PQS activate the Pqs receptor (named MvfR), leading to further QS regulation [35]. Pqs system controls biofilm development and virulence factors production together with the transcription of RhlI (thus indirectly modulating the Rhl-dependent phenotypes) and RhlR activity [35,89,90,91,92]. Moreover, PQS chelates iron and can induce the expression of genes for the biosynthesis of the siderophores pyoverdine and pyochelin for bioavailable iron acquisition [93,94,95].

The fourth QS system, named IQS, is less characterized; the autoinducer 2-(2-hydroxyphenyl) thiazole-4-carbaldehyde controls the activation of Pqs and Rhl systems and bacterial virulence, particularly under phosphate starvation conditions. The synthesis of the autoinducer has been initially associated with the ambBCDE gene cluster and then with a derivative of an intermediate of pyoverdine biosynthesis (pch gene cluster); on the other hand, the receptor identity is still elusive [96,97].

**Figure 3:**
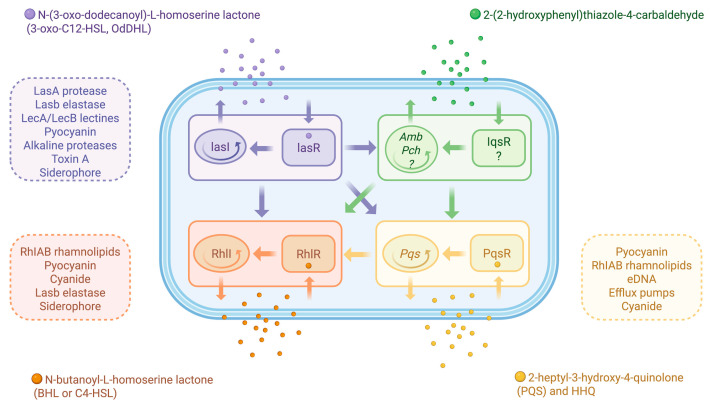
Hierarchical organization of QS machinery in *P. aeruginosa*. Arrow circle indicates the autoinducer synthase activity. Italics show the operons required or supposed to synthesize the autoinducer. The main effects on cell behavior are summarized in the dotted boxes.

A summary of the integrated network of QS systems in *P. aeruginosa* is reported in Figure 3. Upstream Las/Rhl systems, the catabolite repressor homolog Vfr and RelA, which synthesizes the alarmone (ppGpp) under amino acid starvation; Vfr induces LasR expression while RelA induces multiple QS readouts, including Las and Rhl systems [98]. This intricate network of regulation indicates that *P. aeruginosa* integrates demographic information with the nutritional status to finely tune the community response.

### 3.3. QS Inhibition as a Potential Anti-Pathogenicity Strategy Against P. aeruginosa

The great success of QS biosignaling resides in its tentacular control and modulation of many phenotypes relevant to pathogenicity; therefore, targeting QS represents a promising and desirable strategy to tackle *P. aeruginosa*-mediated infections. Possible drugs aimed at interfering with QS are supposed to attenuate pathogenicity, rather than to target bacterial growth or viability. Such molecules, named anti-pathogenic drugs, may represent an innovative strategy to fight the deleterious effects of bacterial colonization, by-passing the risk to sustain the problem of increasing antibiotic-resistance [99]. Moreover, beyond their possible anti-pathogenic effect, QS modulators show multifaceted applicative potential, including anticancer treatment, multifunctional antibacterial materials, chassis-based biosensors and prevention of biofouling [100].

Targeting QS ameliorates the outcome of *P. aeruginosa* infections in animals and cellular models, including *Caenorhabditis elegans* (*C. elegans*), *Dictyostelium discoideum* (*D. discoideum*), *Arabidopsis thaliana* (*A. thaliana*), and murine wound healing and lung infection models [101,102,103,104,105].

**Figure 4:**
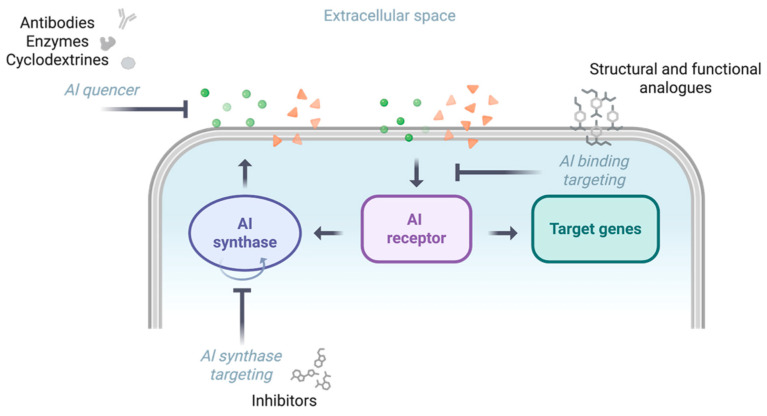
Key strategies for QS inhibition. (i) Degradation or sequestration of autoinducer (AI), aimed at decreasing AI availability required to reach the “quorum”; (ii) inhibition of AI biosynthesis by targeting the proper synthase; (iii) antagonism of AI receptor, to limit the AI-mediated regulation transcription. Icons in the figure refer to antibodies, engineered enzymes, and cyclodextrins, which are used to degrade/trap the AI, while small molecules are obtained by a rational approach or screening (including the virtual one) of chemical or natural libraries of compounds.

Inhibition strategies include targeting signal synthesis or reception, and degrading QS signals enzymatically [106] (Figure 4). Autoinducer quenching can also be achieved by using cyclodextrins able to encapsulate the signal molecules or antibodies sequestering autoinducer molecules, thus leading to reduced AI bioavailability [107,108]. Promising small molecules have been selected by multiple approaches, including natural compounds, synthetic inhibitors, and engineered enzymes (lactonases, acylases and oxidoreductases), with the natural compounds being the main source [109]. Although the Las system is positioned at the top of hierarchical *P. aeruginosa* QS networks, the Las pathway is often inactivated in chronic CF infections, with Rhl and Pqs working autonomously. Therefore, since the Las system could be non-essential in chronic infections, many efforts have been made to also target the Rhll and Pqs systems [109].

## 4. Uropathogenic *E. coli* (UPEC)

### 4.1. Antibiotic Tolerance and Resistance Mechanisms in UPEC

The global burden of urinary tract infections (UTIs), with an estimated 150–250 million cases annually and over 80% caused by UPEC, underscores the critical importance of understanding the mechanisms underlying antibiotic resistance and developing novel therapeutic strategies [20]. Consequently, research into the biological mechanisms of antibiotic tolerance in UPEC has emerged as an important area of research over the past decades. Studies have documented the evolution of UPEC resistance mechanisms, emphasizing the roles of efflux pumps, biofilm formation, and genetic mutations in antibiotic tolerance [15,110]. Yet, the molecular determinants linking biofilm phenotypes to resistance gene expression are debated, and the specific interplay among efflux pumps, biofilm formation, and genetic mutations in conferring antibiotic tolerance in UPEC remains incompletely understood [17,57,111,112]. Conflicting evidence exists concerning the relative contributions of these mechanisms and their temporal dynamics during infection and antibiotic exposure [110,113]. This lack of clarity hinders the advancement of targeted therapies and the implementation of effective antimicrobial stewardship strategies [21].

Multiple studies robustly demonstrate the central role of efflux pumps, particularly the AcrAB-TolC system, in mediating multidrug resistance and antibiotic tolerance in UPEC. Genomic analyses reveal mutations in regulatory genes (e.g., marR, acrR) that upregulate efflux pump expression, contributing to resistance against fluoroquinolones and beta-lactams [114,115,116,117]. Functional assays and inhibitor studies further validate efflux activity as a key resistance mechanism [118]. The identification of efflux pump gene overexpression correlating with clinical resistance phenotypes strengthens the clinical relevance of these findings [117]. Despite extensive characterization, many studies rely heavily on gene presence and expression data without fully elucidating the biochemical or structural dynamics of efflux pump function. Moreover, the majority of studies focus on in vitro or laboratory-evolved strains, limiting understanding of efflux pump regulation in vivo during infection [112].

The causal relationships between biofilm formation and specific resistance mechanisms—such as efflux pump regulation—remain incompletely understood. Notably, some studies report inverse correlations between biofilm formation and antimicrobial resistance [119]. Several investigations underscore the synergistic roles of efflux pump activity, biofilm formation, and genetic mutations in conferring robust antibiotic tolerance. Efflux pumps appear to contribute not only to antibiotic extrusion but also to biofilm development and stability, suggesting a dual role in resistance and persistence. The frequent coexistence of resistance genes and biofilm-forming capacity in clinical isolates highlights the clinical significance of this interplay [111,120,121]. Despite the recognition of these interactions, mechanistic studies dissecting their regulatory crosstalk and temporal dynamics remain limited. Most investigations address these mechanisms in isolation, thereby restricting a comprehensive understanding. Variability in experimental models and strain backgrounds further hampers the delineation of causality.

Whole-genome sequencing and mutation analyses have provided critical insights into the genetic foundations of antibiotic tolerance, identifying key mutations in target genes (e.g., gyrA, parC) and regulatory loci (e.g., marR, acrR) that drive the evolution of resistance [15,114]. Laboratory evolution experiments reveal a temporal progression in which efflux pump overexpression precedes the accumulation of target site mutations, offering a mechanistic framework for resistance development [110]. Furthermore, the identification of compensatory mutations in regulatory genes that activate alternative resistance pathways in efflux-deficient backgrounds highlights the remarkable adaptability of bacterial systems [112].

The frequent co-occurrence of resistance genes and biofilm-forming capability in clinical isolates further emphasizes the clinical relevance of this interplay. Genetic mutations within regulatory networks can simultaneously affect both efflux systems and biofilm-associated genes, potentiating multidrug tolerance [112,115].

Despite recognition of this interplay, mechanistic studies dissecting the regulatory crosstalk and temporal coordination among these factors are scarce. Many investigations treat these mechanisms in isolation, limiting comprehensive understanding. The heterogeneity of experimental models and strain backgrounds further complicates the delineation of causal relationships. Moreover, the influence of environmental and host factors on this interplay remains under-investigated, reducing translational applicability [119,122]. There is a paucity of in vivo studies validating these interactions during infection.

### 4.2. QS Systems in UPEC

**Figure 5:**
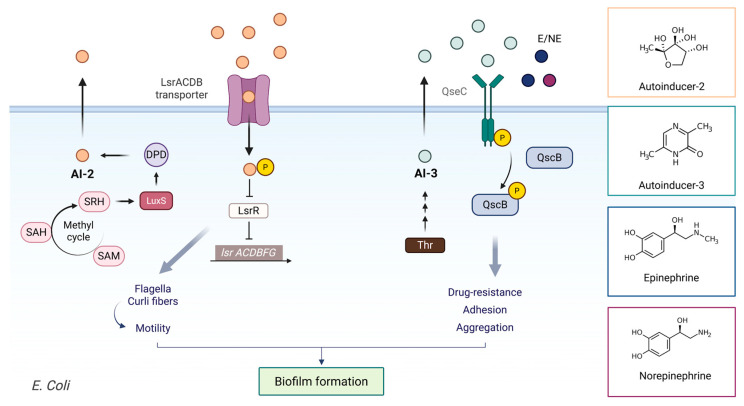
Interconnected signaling pathways in UPEC. Autoinducer-2 (AI-2) mediated quorum sensing and a pathway involving bacterial adrenergic signaling. In the pathway on the left, S-adenosylmethionine (SAM) is converted via S-adenosylhomocysteine (SAH) and S-ribosylhomocysteine (SRH) to 4,5-dihydroxy-2,3-pentanedione (DPD) through the action of the enzyme LuxS. DPD then spontaneously cyclizes to form AI-2, a quorum-sensing molecule released in the milieu and recognized by the LsrABC type transporter. Once internalized and phosphorylated, it (i) releases the LsrR-mediated repression of the Lsr operon, thus allowing further AI-2 import, and (ii) induces the expression of genes involved in drug resistance, aggregation and adhesion. Concurrently, the pathway on the right shows how Autoinducer-3 (AI-3) and the host neurohormones Epinephrine (E) and Norepinephrine (NE) are detected by the membrane-bound sensor kinase QseC. Activation of QseC leads to the phosphorylation of the response regulator QseB. Phosphorylated (activated) QseB, in turn, regulates key processes such as flagellar motility, adhesion, virulence, and biofilm formation. Together, these pathways demonstrate how UPEC integrates signals from its own population density (AI-2 and AI-3) and from the host (E, NE) to coordinate complex physiological and virulence responses, including those processes governing biofilm formation.

QS has emerged as a pivotal mechanism in UPEC, orchestrating virulence, biofilm formation, and antibiotic resistance, key factors underlying the prevalence and recurrence of UTIs. The discovery of QS systems such as LuxS/autoinducer-2 (AI-2) and the AI-3/epinephrine/norepinephrine signaling axis has deepened understanding of bacterial communication and host-pathogen interactions [123,124] (Figure 5).

QS in UPEC primarily relies on the production and detection of AI-2 and AI-3 molecules. Although UPEC lacks a canonical LuxI/LuxR-type AI-1 system and therefore does not synthesize acyl-homoserine lactones (AHLs), it can perceive AHLs produced by other bacterial species through the SdiA receptor [125]. This interspecies “AI-1-like” signaling can influence UPEC biofilm formation and virulence regulation within polymicrobial communities.

The LuxS/AI-2 system constitutes the most extensively characterized cell density-dependent signaling mechanism in UPEC, coordinating bacterial behavior in response to population dynamics and environmental cues. The LuxS enzyme converts S-ribosylhomocysteine (SRH) to homocysteine and 4,5-dihydroxy-2,3-pentanedione (DPD), the precursor of AI-2, which cyclizes into biologically active furanosyl borate diesters [126,127,128]. AI-2 is recognized by the Lsr (LuxS-regulated) system, comprising the transcriptional repressor LsrR, the transporter genes lsrACDBFG, and the kinase LsrK. Accumulated AI-2 is internalized and phosphorylated to AI-2-phosphate, which derepresses lsr transcription, establishing a positive feedback loop [129]. Through regulators such as LsrR, MqsR, and YncC, this system regulates gene expression, controlling motility and biofilm formation.

AI-2 signaling influences UPEC virulence by modulating iron acquisition systems (aerobactin, enterobactin), α-hemolysin production, and acid resistance genes crucial for survival in the urinary tract and gastrointestinal environments [33,124,129,130]. Experimental studies show that AI-2 enhances biofilm biomass and architecture, while luxS disruption impairs multiple virulence traits [33,124,130,131,132,133]. AI-2 analogs and quorum-quenching enzymes targeting this pathway inhibit biofilm maturation and represent promising therapeutic approaches [134,135].

The AI-3 system mediates interkingdom signaling that allows UPEC to sense host catecholamine hormones (epinephrine and norepinephrine) via the QseC sensor kinase, a receptor for both bacterial AI-3 and host neurohormones [123,136]. Upon ligand binding, QseC autophosphorylates and transfers the phosphate to the response regulator QseB, which modulates gene expression [137,138]. The structural similarity between AI-3 and catecholamines enables QseC to respond to both, albeit with different affinities. Host catecholamine signaling enhances motility and metabolic adaptation, whereas AI-3 recognition promotes biofilm maturation and virulence. Exposure to host catecholamines triggers expression of flagellar genes and promotes swimming motility, facilitating dissemination within the urinary tract and potentially contributing to ascending infections [137].

Beyond these canonical systems, UPEC also employs an indole signaling network, representing a distinct form of quorum sensing. Indole, produced by tryptophanase (TnaA), functions as an intracellular and extracellular signal regulating stress response, motility, and biofilm formation [139,140]. Indole modulates the expression of virulence genes through regulators such as SdiA, MqsR/MqsA, and BhsA, often interacting with the AI-2 pathway to fine-tune biofilm development and antibiotic tolerance [141,142]. In UPEC, indole signaling enhances survival under hostile conditions, contributing to persistence in the urinary tract and tolerance to antimicrobial stress. Thus, the indole system represents an additional, metabolically linked communication layer complementing the AI-2 and AI-3 networks in UPEC pathogenesis.

Despite advances, critical gaps remain regarding how LuxS/AI-2 and AI-3/catecholamine systems modulate UPEC biofilm formation and virulence in distinct environments, such as the urinary tract and catheter-associated infections [138,143]. While AI-2 acts as a universal interspecies signal influencing motility and biofilm architecture, the interplay with AI-3 and host adrenergic hormones in UPEC pathogenesis remains incompletely defined [130,144]. Debate persists on whether these QS systems act independently or synergistically in regulating biofilm maturation and antibiotic tolerance. Some studies emphasize AI-2’s metabolic role, whereas others focus on AI-3/QseC-mediated interkingdom signaling [137,145]. This ambiguity hampers the clinical translation of quorum-sensing inhibitors (QSIs), despite their promise [146].

### 4.3. Therapeutic Interventions Targeting QS and Biofilms in UPEC

The central role of the AI-2/LuxS system in UPEC pathogenesis has significant clinical implications, particularly in understanding treatment failures and recurrent infections. Considering the role of altered AI-2 signaling patterns to antibiotic tolerance, through reduced metabolic activity and enhanced matrix production [122], QS inhibitors have started to be investigated as adjunctive therapies that could enhance antibiotic efficacy by disrupting biofilm formation and virulence factor expression [130]. The development of AI-2 antagonists or LuxS enzyme inhibitors could provide novel therapeutic strategies that target bacterial communication networks without directly affecting bacterial viability, potentially reducing selective pressure for resistance development while maintaining the beneficial urogenital microbiota.

Emerging therapies focus on QSIs, including natural compounds like allicin and synthetic molecules such as furanones and LED209, which impair AI-2 and QseC signaling to reduce biofilm formation, motility, and virulence without exerting selective pressure on bacterial growth. Mutational analyses of qseC and qseB, as well as pharmacological inhibition using compounds like LED209, demonstrate significant attenuation of UPEC virulence and biofilm development, reinforcing QseC as a viable therapeutic target [145,147,148]. Clinical and experimental studies have shown variable efficacy of these agents in disrupting UPEC biofilms and enhancing antibiotic susceptibility [146,149,150,151,152]. However, their long-term impact on resistance dynamics and host microbiota remains to be fully elucidated.

The AI-3/catecholamine signaling axis also has significant clinical relevance, particularly in understanding UTI pathogenesis in patients with elevated stress hormone levels or those receiving catecholamine therapy. Critically ill patients or individuals with autonomic dysfunction may experience altered UPEC virulence patterns due to dysregulated catecholamine signaling [138]. Furthermore, the AI-3 system represents a potential therapeutic target for anti-virulence strategies. QseC antagonists or compounds that interfere with AI-3 signaling could potentially attenuate UPEC pathogenicity without exerting direct antimicrobial pressure, thereby reducing the likelihood of resistance development.

## 5. Emerging Trends in Bioinformatics for QS Research

### 5.1. Bioinformatics and Artificial Intelligence in Natural Product Discovery

The complexity and specificity of QS circuits offer multiple targets for intervention. In silico approaches have proven invaluable in identifying QS components, predicting molecular interactions, and modeling signaling networks. These computational strategies can accelerate the discovery of QSIs, guiding experimental validation and therapeutic development. Moreover, in recent years, there has been growing interest in plant-derived natural products as sources of QSIs and biofilm inhibitors. Many phytochemicals can interfere with bacterial signaling or biofilm development, reducing pathogen virulence and enhancing treatment outcomes [153,154,155].

Advances in bioinformatics are reshaping how QS systems are studied, offering novel approaches to understanding and targeting bacterial communication pathways. Computational modeling can predict the pharmacokinetic and toxicological properties of compounds early in the discovery process, streamlining the selection of candidates for further experimental validation [156]. The field of computational drug discovery has evolved significantly, employing a diverse array of methods to accelerate the identification of potential therapeutic compounds. Virtual screening utilizes 3D protein structures to predict ligand binding, while ligand-based approaches leverage known active compounds to identify similar molecules. Molecular dynamics simulations and quantitative structure-activity relationship (QSAR) models provide insights into drug-target interactions and structure-activity relationships. Network pharmacology and chemoinformatics enable the analysis of complex biological systems and large chemical databases. These methods have led to notable successes in antimicrobial discovery, such as the identification of halicin, and have significantly reduced time and costs in early-stage drug discovery [13,157,158,159]. However, limitations persist, including dependence on high-quality training data, challenges in modeling complex biological interactions, and difficulties in accurately predicting pharmacological properties. The integration of machine learning, particularly deep learning, has further enhanced bioactivity prediction capabilities and is pushing the boundaries of in silico drug discovery [160,161,162,163]. Despite challenges, the combination of these computational methods with natural product research offers significant advantages over traditional screening methods for identifying bioactive compounds in plants.

### 5.2. Virtual Screening and Structure-Based Strategies for Targeting QS

Targeting QS systems through in silico methods has emerged as a powerful approach for discovering novel anti-virulence agents that attenuate bacterial pathogenicity without promoting resistance. Among these methods, virtual screening (VS), supported by advances in protein structure prediction and molecular dynamics, plays a central role in identifying and optimizing QSIs, particularly from plant-derived natural compounds.

The integration of these computational approaches allows researchers to simulate, at the atomic level, how small molecules interact with key QS proteins such as LasR, RhlR, LuxS, or QseC, and to predict how these interactions might disrupt bacterial communication, biofilm formation, and virulence regulation.

The molecular architecture of QS systems comprises distinct protein families, including signal synthases, transcriptional regulators, membrane-bound histidine kinases, and transporters, each playing a crucial role in detecting, transducing, and responding to QS signals as described in paragraph 2. These proteins share common structural features that define their function, including ligand-binding pockets, DNA-binding domains, transmembrane helices, and enzymatically active sites, which undergo conformational changes upon interaction with autoinducers or other regulatory elements. Many QS regulators, such as LuxR-type transcription factors, exhibit intrinsic flexibility, transitioning between inactive and active states upon ligand binding, while histidine kinases involved in peptide-based QS rely on transmembrane signaling domains coupled to cytoplasmic kinase and response regulator domains [164]. Beyond Protein–Ligand Interactions (PLIs), Protein–Protein Interactions (PPIs) are central to QS regulation, mediating receptor dimerization, signal transduction, and transcriptional activation. LuxR-like transcription factors often require dimerization or tetramerization upon ligand binding to achieve DNA recognition, while two-component histidine kinase systems undergo phosphotransfer cascades via transient protein complexes that activate response regulators [165,166].

**Figure 6:**
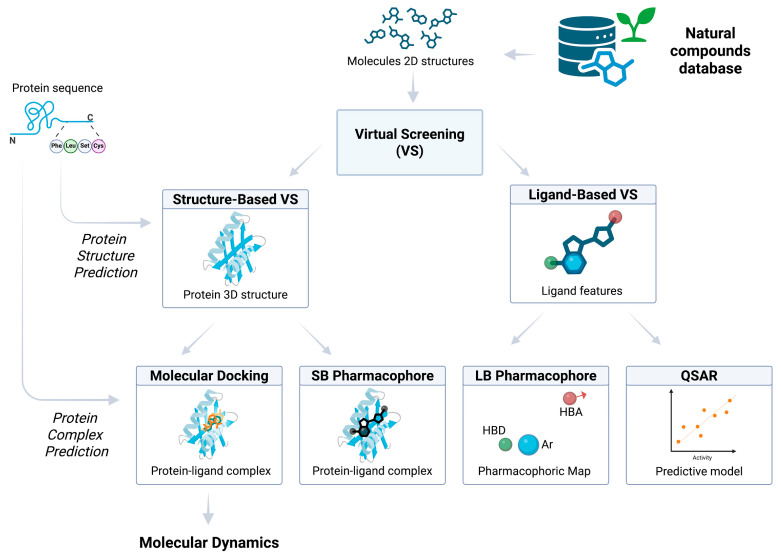
Workflow of virtual screening for natural compounds. This schematic illustrates the key computational approaches for identifying bioactive natural compounds through virtual screening (VS). Molecules from natural compound databases are represented as 2D structures and serve as input for the vs. process, which can follow two complementary strategies: structure-based virtual screening (SBVS), relying on the 3D structure of the target protein, and ligand-based virtual screening (LBVS), based on known bioactive ligand features. Protein structure prediction provides 3D models used in SBVS, followed by molecular docking and structure-based pharmacophore (SB pharmacophore) modeling to identify key binding interactions. LBVS approaches include ligand-based pharmacophore (LB pharmacophore) modeling and quantitative structure–activity relationship (QSAR) analysis to generate predictive models correlating structure with activity. Predicted protein–ligand complexes are refined through molecular dynamics simulations to assess binding stability and conformational flexibility, supporting hit validation and lead optimization.

Given the dynamic nature of these interactions, ranging from transient ligand binding to stable protein complexes, structural bioinformatics tools such as molecular docking, molecular dynamics simulations, and PPI modeling are essential for predicting their behavior (Figure 6). The structural characterization of these complexes, whether through experimental approaches like X-ray crystallography and cryo-electron microscopy or computational modeling techniques, is crucial for understanding their molecular function and for the rational design of QSIs.

#### 5.2.1. Protein Modeling for QS Targets

Accurate 3D structures of QS-related proteins are essential for structure-based drug design and virtual screening. Traditionally, structure prediction has relied on template-based modeling, which leverages the structural information of homologous proteins as templates. This homology-based approach assumes that proteins sharing significant sequence similarity adopt similar folds. Well-established tools such as SWISS-MODEL [167], Phyre2 [168] and MODELLER [169] exemplify this methodology, utilizing experimentally resolved structures from repositories like the Protein Data Bank (PDB) to generate models of proteins lacking resolved structures. For example, the LuxS enzyme in *K. pneumoniae* or *E. coli*, which is involved in AI-2 quorum sensing and biofilm formation that lacks an experimentally resolved structure, could be studied through its close homolog from *Salmonella enterica* (*S. enterica*), whose LuxS proteins have been experimentally solved (PDB-ID:5E68). However, template-based methods are inherently limited when homologous templates are unavailable, especially for proteins from less-studied organisms or those exhibiting significant sequence divergence. Recent advances in deep learning have now revolutionized the landscape of protein structure prediction.

Novel ab initio methods, which predict protein folds without the need for homologous templates, now achieved atom-level accuracy by integrating co-evolutionary coupling analysis from multiple sequence alignments, physical energy potentials and deep neural networks with transformer architectures (e.g., AlphaFold2, RoseTTAFold) [170,171]. Notably, AlphaFold 2 (AF2) has set a new benchmark in the field by delivering near-experimental resolution models even for proteins lacking known structural homologs, thereby significantly expanding the scope of structure-based functional insights into QS systems [170]. *P. aeruginosa*’s QS systems feature a combination of experimentally determined and AF2-predicted structures. For example, while LasI and LasR (components of the Las system) have experimentally resolved structures, RhlI is characterized by AF2-predicted models only, which have proven valuable for mapping clinically relevant mutations and understanding their impact on enzyme function [172].

While AF2 achieves near-experimental accuracy for single-domain protein prediction, its performance remains limited for multi-domain proteins, which are prevalent in QS systems, such as histidine kinases and LuxR-type transcription factors. These proteins often consist of distinct functional domains, such as ligand-binding, transmembrane, and catalytic domains, that exhibit varying degrees of flexibility and interaction. Template-based methods excel at predicting well-conserved domains with available homologs, while ab initio methods like AF2 can accurately model orphan or highly divergent domains. However, neither approach alone is sufficient for capturing the full complexity of multi-domain proteins, as template-based methods may struggle with domain arrangements that lack homologous templates, and de novo methods may face challenges in accurately predicting inter-domain interactions and conformational dynamics [173]. By integrating both approaches, researchers can leverage the strengths of each method to generate more comprehensive and reliable models, enabling a deeper understanding of the structural and functional mechanisms underlying QS.

QS proteins engage in critical interactions with a variety of biomolecules to orchestrate bacterial communication and regulation. Recent developments in deep learning (DL) frameworks such as AlphaFold3 and RosettaFoldAllAtoms have significantly enhanced the accuracy and scope of modeling, extending beyond individual protein folds to encompass complex assemblies involving protein–protein, protein–ligand, and protein–nucleic acid interactions [174,175]. Although it is still rare to find applications for QS-related proteins. The latest DL-based methods for protein complex prediction hold significant promise for advancing our understanding of such mechanisms across diverse bacterial systems. For example, in *P. aeruginosa*, such methods could be used to predict the interaction interfaces between LasR and RhlR, two LuxR-type transcription factors that coordinate the expression of virulence and biofilm genes. For protein–ligand interactions, DL-driven modeling could elucidate how the autoinducer *N*-acyl homoserine lactone (AHL), synthesized by LuxI in *V. fischeri*, binds to the LuxR receptor to activate bioluminescence [176,177]. Finally, for protein–nucleic acid complexes, these approaches could predict how LuxR-DNA binding occurs in *V. fischeri*, providing insight into the regulation of the lux operon. By enabling accurate modeling of these diverse interactions, artificial intelligence methods have the potential to reveal the structural basis for QS signal integration, specificity, and regulatory control across both Gram-negative and Gram-positive bacteria.

#### 5.2.2. Virtual Screening of Anti-QS Natural Compounds

Among the computational approaches advancing QS-targeted drug discovery, vs. stands out for its efficiency in identifying QSIs from large compound libraries. Simulating interactions between small molecules and key QS targets vs. enables the rapid identification of compounds that can competitively inhibit signal binding, block receptor activation, or interfere with gene expression cascades, thereby disrupting bacterial communication [178,179]. vs. strategies are typically divided into structure-based virtual screening (SBVS) and ligand-based virtual screening (LBVS), each offering distinct advantages in the context of QS. SBVS leverages the 3D structures of QS targets—such as AHL synthases (e.g., LasI), receptors (e.g., LasR, LuxR homologs), or AI-2 processing enzymes—to evaluate how the candidate molecules fit into their active or allosteric sites, predicting binding affinity and mode of interaction. In SBVS, molecular docking is commonly used as a complementary approach to evaluate how well compounds fit into the binding site of a QS target [180]. Docking algorithms apply scoring functions that account for key physicochemical interactions—such as hydrogen bonding, hydrophobic contacts, electrostatics, and steric complementarity—to estimate binding affinity [181]. This process also aids in exploring the molecular basis of inhibition by revealing how candidate molecules may disrupt signal recognition or receptor activation. In contrast, LBVS relies on the known activity of existing QSIs, using their pharmacophoric features or QSAR models to identify structurally similar compounds.

The chemical diversity found in plant-derived metabolites offers a rich source of potential QSIs, many of which are less likely to induce resistance compared to synthetic antimicrobials. Recent studies have demonstrated the efficacy of vs. in identifying natural compounds as QSIs. For instance, a SBVS of over 2500 phytochemicals from Indian medicinal plants against QS targets in *P. aeruginosa* and *Chromobacterium violaceum* identified flavonoids such as quercetin, rutin, and baicalein as top candidates based on docking scores and drug-likeness; these findings were further supported by molecular dynamics simulations and ADMET profiling, which evaluates a compound’s absorption, distribution, metabolism, excretion, and toxicity [182]. Similarly, the screening of 186 compounds from Stachys species against six QS proteins in *P. aeruginosa* (LasI, LasR, RhlI, RhlR, PqsA, and PqsR), identified chrysosplenetin, syringic acid, and vanillic acid as potent QSIs with favorable pharmacokinetic properties [183]. Rajamanikandan et al., 2017, employed a combination of molecular docking, molecular dynamics simulations, and e-pharmacophore modeling to investigate the binding mode of cinnamaldehyde derivatives with *Vibrio harveyi* (*V. harveyi*) LuxR, a master transcriptional regulator of QS [184]. The study identified a potent QSI through shape and e-pharmacophore-based virtual screening. In vitro assays demonstrated that this compound significantly reduced biofilm formation and motility in *V. harveyi* in a dose-dependent manner. A classic example of this approach is the study by Zeng et al., 2008 [185], in which DOCK 5.3.0 was used to screen 51 bioactive components from Traditional Chinese Medicines for QS inhibition in *P. aeruginosa*. Baicalein was identified as a promising QSI of biofilm formation without affecting bacterial growth [185,186]. Notably, baicalein also enhanced proteolysis of the TraR receptor protein in *E. coli*, highlighting its potential as an anti-virulence agent. To enhance reproducibility and broader application of these methods, dedicated protocols are increasingly being published. Fernandes et al., 2024, for example, provided a practical pipeline for the in silico screening of phytochemicals targeting LuxS and LasR, incorporating docking, scoring, and toxicity filtering steps [187].

While the above studies relied on small, manually curated sets of phytochemicals, a growing number of publicly available databases now offer broader access to diverse, annotated natural products. A recent review by Zeng et al., 2024, summarizes a wide range of such resources, including examples like COCONUT and SuperNatural 3.0, which provide extensive compound libraries with associated bioactivity, structural, and pharmacokinetic data—enabling large-scale vs. campaigns [188,189,190].

Although not focused on natural products, several studies illustrate complementary vs. strategies that could be adapted to phytochemical screening. Docking-based studies have provided valuable insights into the design of QSIs. For instance, iterative docking and scoring approaches identified triazole derivatives AI-2 competitors in *Salmonella*, effectively mimicking the natural AI-2 signal and disrupting QS-mediated behaviors [191]. These findings demonstrate the utility of docking tools in identifying and optimizing QSIs, particularly for targeting key ligand–receptor interactions in diverse bacterial species. Another study applied vs. to identify inhibitors targeting LasR in *P. aeruginosa*, revealing several compounds with strong binding affinities and inhibitory effects on biofilm formation and virulence factor production [192].

#### 5.2.3. Molecular Dynamics for Hit Validation

Molecular dynamics (MD) simulations are powerful computational techniques that model the physical movements of atoms and molecules over time, providing dynamic, atomistic insights into biomolecular systems. Rooted in Newtonian mechanics, MD simulations calculate atomic trajectories by solving equations of motion based on predefined force fields such as AMBER, CHARMM, or GROMOS [193,194,195]. Unlike static modeling approaches, MD enables the exploration of conformational flexibility, binding stability, and dynamic molecular interactions, making it indispensable for drug discovery. MD platforms such as GROMACS and AMBER allow researchers to simulate the time-dependent behavior of QS receptors upon ligand binding [193,196].

For instance, MD simulations combined with the Molecular Mechanics Generalized Born Surface Area (MM-GB/SA) method were employed to study the LuxR QS protein, identifying key residues critical for signal sensitivity. This approach enabled the rational engineering of LuxR-family proteins for biosensor applications, demonstrating the utility of MD in designing bioengineering tools [197]. MD simulations are particularly valuable for refining the binding modes of QSIs identified through vs. or molecular docking. They help validate docking predictions by assessing the stability of ligand–QS receptor complexes over time, while also revealing key molecular interactions, potential allosteric effects, and induced-fit conformational changes [198]. For instance, the study from Chaieb et al., 2022, already mentioned as an example of vs. of natural compounds, also employed MD simulations to evaluate the 19 natural compounds [182]. Catechin and nakinadine B were identified as potent QS antagonists, showing stable interactions and favorable binding energies during a 50-nanosecond simulation (e.g., root mean square deviation analysis). Integrated molecular dynamics and virtual screening approaches facilitated the discovery of QSIs targeting LsrK, a key regulator of the AI-2 signaling pathway, with functional validation in cell-based assays confirming their potential to disrupt bacterial communication and biofilm formation [199].

Beyond identifying inhibitors, MD simulations enable quantitative insights into binding free energies using methods such as MM-PBSA or free energy perturbation (FEP). These calculations aid in optimizing lead compounds for improved binding affinity and specificity. For instance, MD-based free energy calculations have been applied to prioritize QSIs targeting AI-2 receptors, facilitating the discovery of novel inhibitors with potential applications in controlling biofilm formation and bacterial virulence [200]. Similarly, computational workflows integrating MD have been used to study the inactive form of SdiA in *E. coli*, providing insights into its regulatory mechanism under QS-inhibitory conditions [201]. Recent advancements also include combining MD with terahertz spectroscopy for intelligent matching of quorum signal molecules like *N*-acyl-homoserine lactones (AHLs). This integration has enabled precise identification of AHLs through a two-step similarity matching method, showcasing the potential of MD in enhancing experimental approaches for QS research [202].

### 5.3. Methodological Reproducibility in Computational QS Research

The expanding application of bioinformatics, molecular modeling, and machine learning approaches in QS and antibiofilm research necessitates careful consideration of methodological reproducibility to ensure robustness, interpretability, and translational relevance of computational findings. General guidelines for reproducible computational research emphasize that reliability critically depends on data quality, transparent reporting of workflows, and explicit documentation of algorithmic parameters [203,204]. In the context of QS-targeted studies, this includes the use of well-curated protein sequences and chemical libraries, confidence-assessed experimental or predicted protein structures, and standardized preparation of ligands and binding sites. Structural bioinformatics studies have shown that variations in docking software, scoring functions, grid definitions, protonation states, or force fields can substantially affect predicted protein–ligand interactions, underscoring the need for detailed methodological reporting and benchmarking [205]. Similar recommendations apply to molecular dynamics simulations, where simulation length, convergence criteria, and force-field selection influence stability and binding-energy estimates [206]. Moreover, machine learning approaches increasingly used to predict QS inhibitors are particularly sensitive to training dataset composition, feature selection, and validation strategies, raising well-recognized reproducibility concerns in data-driven drug discovery [207]. In line with these best-practice recommendations, the adoption of standardized, openly described computational workflows and benchmark datasets would substantially improve cross-study comparability. Importantly, the development of shared, community-accessible predictive models and virtual screening pipelines for conserved QS-related protein targets could further enhance reproducibility, facilitate independent validation, and accelerate the discovery of reliable QS inhibitors with genuine translational potential.

To support transparency and facilitate reproducible implementation of these approaches, we provide Table 1, which summarizes a representative selection of widely used, free, and open-source tools for virtual screening, molecular docking, and molecular dynamics simulations commonly employed in drug discovery workflows. While many of the practices currently applied in QS research, such as protein structure prediction, molecular docking, molecular dynamics simulations, and machine learning, are derived from general computational drug-discovery guidelines rather than QS-specific frameworks, the availability of shared tools and openly accessible software already offers a solid technical foundation. Widely used community resources, including public structural repositories, natural product databases, and open-source docking or simulation tools, already provide a solid technical foundation. Even though several individual studies and protocols have proposed reproducible in silico workflows for targeting specific QS proteins, at present, there are no broadly adopted, community-wide predictive models or standardized virtual screening pipelines explicitly dedicated to QS-related protein targets, nor shared benchmark datasets analogous to those available in more established drug-discovery areas [208]. Therefore, while QS research benefits from existing general best practices in computational biology, there is a clear opportunity and need for the development of QS-focused shared resources, including curated target sets, reference ligands, standardized workflows, and openly accessible predictive models, to improve reproducibility and accelerate translational progress.

## 6. Identifying Natural and Synthetic QS Inhibitors

### 6.1. Ethnobotanical Approaches to Selecting Medicinal Plants

One fruitful strategy for discovering anti-QS and anti-biofilm agents is ethnobotanical selection, based on traditional medicinal knowledge, which helps identify plants historically used to treat infectious conditions [155,237].

Researchers often begin by analyzing ethnomedicinal records and selecting plant species known for antimicrobial or wound-healing uses in traditional practice. These “ethnobotanicals” are likely to contain bioactive metabolites with potential QS-disrupting activity. Knowledge-driven selection significantly increases the hit rate for finding anti-virulence botanicals compared to random screening approaches. Ethnobotanical surveys across different regions such as Europe, Africa, Asia, Latin America have identified numerous candidate plants whose extracts show QSI activity in lab tests. Plants traditionally used to treat infections or inflammation tend to be enriched in metabolites with QS or biofilm inhibitory potential [238,239].

To detect and analyze bacterial QS signals, scientists employ mass spectrometry, chromatography, biosensor-based, immunochemical, and analytical chemical techniques. These approaches support antibiotic development by providing diverse sensitivity ranges and versatile applications [240].

Once candidate plants are selected, standard natural product extraction techniques are employed to obtain the crude phytochemical mixtures. The plant material (leaves, flowers, roots, seeds, bark and others) is usually dried, ground, and extracted with suitable solvents to dissolve secondary metabolites. Common extraction methods include maceration (cold or room-temperature soaking), decoction/infusion (hot water or solvent extraction) and Soxhlet extraction (continuous reflux extraction with a solvent). In many studies, a sequential extraction is performed, using solvents of increasing polarity to obtain fractions enriched in chemically distinct metabolites [241].

**Figure 7:**
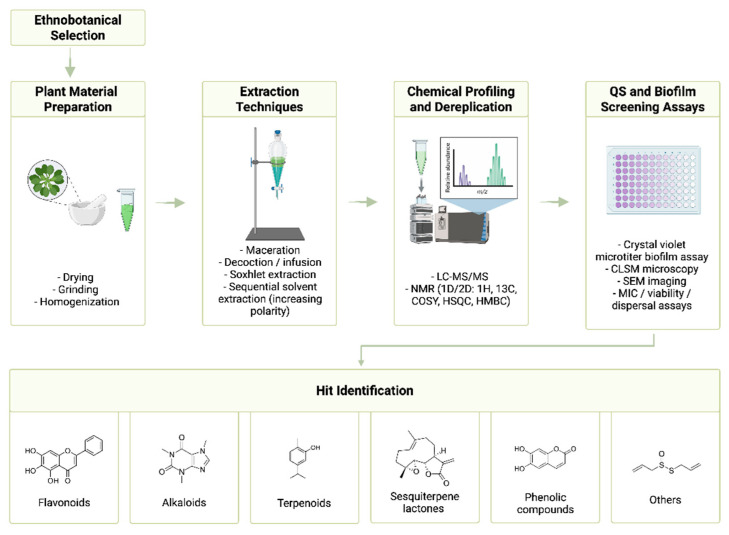
Workflow for identification and characterisation of QSIs and antibiofilm compounds from medicinal plants. Ethnobotanical knowledge guides plant selection, followed by material preparation and extraction using methods such as maceration, decoction, or Soxhlet extraction. Extracts are profiled by Liquid chromatography tandem mass spectrometry (LC-MS/MS) and nuclear magnetic resonance (NMR) for chemical identification, characterization and dereplication, then screened using QS and biofilm assays (e.g., crystal violet microtiter assay, CLSM, SEM, MIC/viability tests). This integrated approach enables the identification of active phytochemical classes, including flavonoids, alkaloids, terpenoids, sesquiterpene lactones, and phenolic compounds.

The resulting liquid–liquid partitioning step suspends the crude extract in water and separates it with immiscible solvents (n-hexane, ethyl acetate, or n-butanol), according to solubility differences. Each solvent fraction, non-polar (hexane), moderately polar (ethyl acetate), or highly polar (aqueous), can then be screened individually for bioactivity, enriching the active constituents and simplifying subsequent isolation [241] (Figure 7).

### 6.2. Plants as Resources of Novel Antibiotics

Plant-derived compounds have long played a crucial role in the development of medicinal therapies. They produce a wide variety of bioactive chemical compounds, such as alkaloids, flavonoids, terpenoids, and polyphenols, which they use as defense mechanisms against pathogens like bacteria, fungi, and viruses. Many of these compounds possess antimicrobial properties and have been studied and are in use in treating AMR [18,153,154,162,242,243,244,245,246].

Their exploration not only highlights the importance of the biodiversity of plant species but also emphasizes the importance of traditional medicine and natural products in modern drug discovery. Only a small fraction of plant species has been systematically investigated for chemical compounds with antibiotic potential. Recent advances in bioinformatics, machine learning, and natural product chemistry offer exciting new opportunities to accelerate the discovery of plant-derived antibiotics. In the context of the One Health approach, it is important to mention that the plants represent a renewable resource for drug discovery. This aligns with the One Health goal of finding sustainable solutions to health challenges that do not negatively impact ecosystems [247].

The so-called “traditional approach” in natural product chemistry and antimicrobial testing typically involves a sequential process starting with plant selection based on ethnopharmacological knowledge or random screening, which can very often be hit-or-miss. This is followed by the extraction of plant material using various solvents and techniques. The resulting extract is exposed to chemical analysis that employs powerful analytical techniques like gas chromatography, (ultra) high-performance liquid chromatography, and liquid chromatography (LC) combined with mass spectrometry (MS). This would result in the identification and characterisation of the number of compounds present in the plant extract. The techniques used would depend on the chemical properties of the isolated extracts and chemical compounds researchers wish to study, going from volatile to non-volatile chemical compounds. Following the natural product chemical characterisation, antimicrobial screening of these extracts and fractions would usually be conducted using methods such as disk diffusion assays and broth microdilution to determine MIC on a set of microbes. This would provide insight into the potential of plant extracts as a source of new antibiotics [155,248,249,250,251,252,253,254].

Traditional methods for discovering plant-based antimicrobial compounds have several significant drawbacks. First, the process is highly time-consuming and resource-intensive, requiring substantial laboratory work and expensive equipment. Extracting, analyzing, and characterizing compounds from plants can take considerable time, slowing down the discovery process. This approach risks overlooking potentially valuable plant species that could contain effective antimicrobial compounds. The analytical techniques, while powerful, can be biased towards detecting certain types of compounds, potentially missing novel structures or complex mixtures that might have synergistic effects. This approach often focuses on isolating and identifying individual compounds, which may overlook the importance of synergistic interactions between multiple components in a plant extract. The sequential nature of chemical analysis followed by antimicrobial testing means that a large amount of time and resources is spent analyzing compounds that ultimately exhibit little to no antimicrobial activity, leading to inefficiency in the discovery process.

Researchers frequently attempt to link antimicrobial activity with the dominant constituents of plant extracts, yet the complexity of these mixtures often obscures which metabolites drive the observed effects. To address this, a range of chemometric and multivariate approaches, including PCA and related dimensional-reduction or clustering methods, have been applied to associate chemical profiles with bioactivity [255,256]. Complemented by advances in bioinformatics and machine-learning models, these methods now enable the prediction of antimicrobial potential directly from structural features of plant-derived compounds. However, this approach was very often limited to in silico modelling and theoretical explanations, without testing on bacteria, either fractions or pure compounds, to confirm statements.

Plant-derived compounds with anti-QS and anti-biofilm activity are typically elucidated using MS and nuclear magnetic resonance (NMR) as complementary techniques. Mass spectrometry provides critical information like molecular weight and formula (especially via high-resolution MS) and generates fragmentation patterns (through tandem MS/MS) that hint at substructures. Liquid chromatography–MS workflows (LC-MS/MS) allow for the identification of bioactive constituents from plant extracts by their unique mass spectra. The NMR spectroscopy, in turn, reveals the detailed atomic connectivity and functional groups: one-dimensional ^1H and ^13C NMR spectra define the types of protons and carbons present, while two-dimensional experiments (COSY, HSQC, HMBC, etc.) correlate nuclei to map out the molecule’s skeleton. By combining MS with 1D/2D NMR data, the complete structures of isolated natural products can be obtained. This integrated approach is the cornerstone of natural product chemistry, ensuring that new anti-QS or anti-biofilm compounds are accurately identified and differentiated from known substances (dereplication) before biological evaluation.

### 6.3. In Vitro Testing of Biofilm Formation Inhibition

Complementing QS assays, a suite of in vitro biofilm assays is used to evaluate the ability of plant-derived substances to prevent or disrupt biofilms [257]. The most widely used method is the microtiter plate biofilm formation assay with crystal violet staining [258]. Bacteria are grown in 96-well plates with and without the test extract/compound. After incubation, non-adherent cells are washed off and the remaining biofilm biomass is stained with crystal violet dye, which binds to the polysaccharide/protein matrix and cells. The dye is then solubilized and quantified by measuring absorbance, providing a measure of biofilm mass. A significant reduction in crystal violet absorbance in treated wells indicates antibiofilm activity. This assay is straightforward and high-throughput, making it a standard tool for initial screening. Variations in this method use other stains like safranin, but the principle remains the same. Researchers often report percent inhibition of biofilm formation relative to an untreated control. For example, many plant extracts have shown >50% inhibition of biofilm attachment in such assays at subMIC concentrations [259].

Beyond bulk biomass measurements, microscopic techniques are employed to visualize how natural products affect biofilm architecture and viability. Confocal laser scanning microscopy (CLSM) is frequently used in conjunction with fluorescent viability stains (e.g., Syto9/propidium iodide) to observe biofilm structure in three dimensions. Treated biofilms can be compared to controls for thickness, substratum coverage, and live/dead cell distribution. A plant-derived chemical compound that penetrates the biofilm might show more dead (red) cells and a thinner biofilm matrix in CLSM and scanning electron microscopy (SEM) to confirm a reduction in biofilm density and cell clustering [260].

Other complementary assays include measuring the viability of biofilm-encased bacteria (e.g., via plate counts or MTT metabolic reduction assays) and biofilm eradication tests, where pre-formed biofilms are treated with the compound to see if it can break down established biofilms. Reductions in colony-forming units from treated biofilms or increased release of cells can indicate a dispersal effect. In some studies, surface hydrophobicity and exopolysaccharide production are measured, as these factors influence biofilm formation. By using the crystal violet assay for quantification and microscopy for visualization, investigators can assess both the extent and nature of biofilm inhibition caused by plant-derived agents. Combining these with QS assays helps distinguish general anti-bacterial effects from specific anti-biofilm (anti-adhesion or matrix disruption) and anti-QS (anti-virulence) effects.

### 6.4. Phytochemical Classes with Anti-QS and Anti-Biofilm Activity

Plants produce a vast diversity of secondary metabolites, yet certain classes have consistently demonstrated anti-QS and antibiofilm properties. The most common active phytochemicals reported in the last decade include chemical compounds such as flavonoids, alkaloids, terpenoids, sesquiterpene lactones and phenolic compounds [261,262,263]. Representative QS targets, classes of plant-derived compounds, and the computational and experimental strategies employed to investigate these interactions are summarized in Table 2, providing an integrative overview of current discovery pipelines.

#### 6.4.1. Flavonoids

This large family of polyphenolic compounds (e.g., quercetin, naringenin, baicalein) has yielded many QS inhibitors. Flavonoids can antagonize QS receptors in Gram-negatives—for example, some flavones bind to *P. aeruginosa* LasR/RhlR receptors and prevent them from activating QS genes. It was shown that flavonoids with certain structural features (two hydroxyl groups on the A-ring) non-competitively inhibited LasR/RhlR, blocking QS-controlled gene expression and suppressing virulence factor production. Flavonoids such as baicalein (from Scutellaria roots) and quercetin have demonstrated strong antibiofilm activity as well, often in conjunction with QS inhibition [264,265]. 

#### 6.4.2. Alkaloids

Alkaloids are nitrogen-containing basic compounds, many of which show antimicrobial or signaling-interference activity. Several alkaloids from medicinal plants or even dietary sources act as QS quenchers. For instance, hordenine [268], an alkaloid from sprouting barley, was found to inhibit AHL-mediated QS in *P. aeruginosa*, leading to reduced production of multiple QS-regulated virulence factors and biofilm suppression. It downregulated the expression of key QS genes (las and rhl systems) in *P. aeruginosa*, effectively attenuating the pathogen’s virulence without killing it. Other examples include caffeine (a purine alkaloid), which showed antibiofilm effects and interference in *P. aeruginosa* swarming, motility and other virulence factors. Berberine (an isoquinoline alkaloid from plants like Berberis) has been reported to inhibit biofilm formation in Staphylococcus aureus and *P. aeruginosa* (often by direct antimicrobial action coupled with some QS modulation) [269]. Alkaloids are chemically diverse (indoles, quinolines, tropanes, etc.), and interest in them as antivirulence agents remains high.

#### 6.4.3. Terpenoids

Terpenoids (isoprenoids) include monoterpenes, sesquiterpenes, diterpenes, and triterpenes found in essential oils, resins, and plant trichomes. Owing to their lipophilicity and membrane activity, many terpenoids disrupt biofilms or signaling. Monoterpenes such as carvacrol (from oregano) and 1,8-cineole (eucalyptus oil component) can inhibit biofilm formation by *P. aeruginosa* and other bacteria [266]. Carvacrol and its isomer thymol are known to intercalate into bacterial membranes and have been shown to reduce QS-regulated pigment production and biofilm mass in Pseudomonas.

#### 6.4.4. Sesquiterpene Lactones

Parthenolide, e.g., from Tanacetum parthenium, feverfew have also demonstrated QSI activity by inhibiting *P. aeruginosa* PAO1 biofilms and reducing the levels of its 3-oxo-C12-HSL signal molecule [267]. Triterpenoids (C30 compounds) and their saponin derivatives are another important group—pentacyclic triterpenes like ursolic acid and oleanolic acid (widely found in medicinal plants) can inhibit staphylococcal and pseudomonal biofilms. One study found that two triterpenic acids from an ethnomedicinal plant not only disrupted *P. aeruginosa* biofilms in vitro and on medical catheter surfaces, but also downregulated multiple QS genes (lasI, lasR, rhlI, rhlR) in the bacteria. Terpenoids often target bacterial cell envelopes and can modulate signaling pathways, making them versatile antibiofilm agents. Many essential oils rich in terpenoids (e.g., tea tree oil, clove oil) exhibit broad-spectrum biofilm inhibition, though their exact modes of action can involve membrane disruption in addition to any QS effects.

#### 6.4.5. Phenolic Compounds

Plant phenolics are well-known for their antimicrobial and antioxidant properties, and numerous phenolics also interfere with QS and biofilm formation. This broad category includes simple phenols, phenolic acids, polyphenols, coumarins, and tannins. Coumarins (benzopyrone derivatives) have gained attention as natural QS inhibitors. Esculetin (6,7-dihydroxycoumarin) [270,271], for example, was shown to markedly inhibit QS-regulated toxin production and biofilm in *Aeromonas hydrophila* (*A. hydrophila*). It diminished the bacteria’s protease and hemolysin secretion (both QS-controlled virulence factors) and impaired biofilm development, while also downregulating QS regulatory genes. Other coumarins like umbelliferone and coumaric acid derivatives have shown similar QS and biofilm inhibitory effects in Pseudomonas and Chromobacterium models. Often, these are isolated from traditional medicinal plants such as tonka bean or sweet clover. Phenylpropanoids like cinnamaldehyde (from cinnamon bark) and eugenol (from clove) are another subgroup: these have been found to inhibit AI-2-based QS signaling in vibrios and reduce biofilm levels in UPEC and Salmonella biofilms by affecting motility and matrix production. Tannins and other polyphenols (abundant in plant bark and galls) can bind proteins and disrupt bacterial adhesins; indeed, tannic acid has been reported to inhibit *P. aeruginosa* QS signals and biofilm formation in some studies. Overall, phenolic phytochemicals, due to their reactive hydroxyl groups, may oxidatively modify signaling molecules or receptors or interfere with the regulatory networks controlling biofilm formation. Many are also antioxidants, which can mitigate the oxidative stress responses in biofilms.

#### 6.4.6. Other Natural Chemical Compound Classes

Other components also play an important role: organosulfur compounds from plants such as garlic’s allicin and ajoene have demonstrated potent quorum quenching and biofilm dispersion activity. Saponins (glycosylated triterpenes) have been shown to inhibit biofilms by destabilizing the exopolysaccharide matrix. However, the four classes above (flavonoids, alkaloids, terpenoids, and phenolics) encompass the majority of plant-derived anti-QS/anti-biofilm agents reported in the last decade. Often, multiple classes of compounds are present in a single plant extract and may act synergistically to achieve the observed antivirulence effect.

Plant-derived QS and biofilm inhibitors represent a compelling avenue for anti-infective therapy development. Traditional medicinal knowledge guides the selection of promising plants, and advances in extraction and analytical methods enable the discovery of active phytochemicals. By using in vitro QS reporter assays and biofilm models, researchers can pinpoint extracts and compounds that specifically attenuate bacterial communication and community formation without necessarily killing the bacteria. Flavonoids, alkaloids, terpenoids, and phenolics have emerged as key players, often capable of reducing pathogen virulence by targeting QS circuits (e.g., lasR/rhlR in *P. aeruginosa*) or by preventing the establishment of protective biofilms. The examples from recent literature underscore not only the diversity of botanical metabolites with such activities but also the potential for these agents to work in vivo and to synergize with antibiotics. While challenges remain—such as ensuring stability, bioavailability, and safety of these natural products—the progress of the past decade reinforces that nature’s chemical arsenal, honed by evolutionary interactions, is a valuable source of antivirulence compounds. Ongoing interdisciplinary research at the interface of ethnobotany, microbiology, and natural product chemistry will continue to yield insights and lead compounds, hopefully translating into novel therapeutic strategies to mitigate bacterial virulence and biofilm-related infections.

## 7. Clinical Relevance and Translational Constraints of QS-Targeted Strategies

From a translational standpoint, QS inhibition has emerged as an anti-virulence strategy aimed at modulating pathogenic behavior rather than directly suppressing bacterial growth. This conceptual shift underlies the prevailing view that QSIs are unlikely to function as stand-alone therapeutics, but may instead find their most realistic application as adjuncts to conventional antimicrobial treatments [99,272]. In clinical contexts characterized by chronicity and persistence, such as biofilm-associated or device-related infections, QS-regulated coordination of virulence and community behavior plays a central role in therapeutic failure, providing a rationale for interventions that weaken these collective traits and thereby increase susceptibility to antibiotics or host defenses. However, the translation of this rationale into clinical practice has proven challenging. One fundamental obstacle lies in the intrinsic architecture of QS systems themselves: their hierarchical organization, redundancy, and adaptive plasticity enable pathogens such as *P. aeruginosa* to compensate for partial pathway inhibition, limiting the durability of single-target interventions [273]. This adaptive capacity also complicates assumptions regarding resistance, as selective pressures may favor alternative regulatory routes or phenotypic tolerance rather than classical target-site resistance. At the same time, the experimental systems commonly used to evaluate QS inhibition often lack sufficient predictive power for clinical outcomes. Simplified in vitro biofilm and QS assays do not fully capture the spatial heterogeneity, host-derived signals, polymicrobial interactions, and environmental constraints that shape QS activity in vivo, making it difficult to reliably extrapolate experimental efficacy to real infections [274]. The diversity of pathogens, the individual variability in host responses, and the spatiotemporal heterogeneity of nosocomial infections highlight the critical role of pathogen–host interactions in disease progression; this complex scenario demands the identification of (future) specific treatment subtypes and key biomarkers for these subtypes [275]. 

These biological uncertainties intersect with pharmacological limitations, particularly for plant-derived QS inhibitors, many of which exhibit suboptimal solubility, metabolic stability, or bioavailability that restrict systemic administration. As a consequence, effective QS inhibition may depend as much on formulation and delivery strategies—such as localized, device-associated, or surface-based applications—as on intrinsic compound potency. Furthermore, QS activity is highly dynamic and context-dependent during infection, suggesting that therapeutic timing and patient stratification may critically influence outcomes, and that QS inhibition may be beneficial only in specific stages or subsets of disease. Finally, the clinical development of QS inhibitors faces additional challenges related to trial design and regulatory evaluation, as anti-virulence agents do not readily align with traditional endpoints based on bacterial eradication. Taken together, these interrelated factors help explain why QS inhibitors, despite strong mechanistic foundations and extensive preclinical investigation, have not yet transitioned into routine clinical use. While dedicated reviews have explored these translational challenges in greater depth, they are summarized here to situate QS-targeted approaches within a realistic therapeutic framework and to underscore the complexity of bridging QS biology and clinical intervention [276].

## 8. Conclusions

The growing threat of antimicrobial resistance (AMR) underscores the urgent need for therapeutic strategies that extend beyond traditional antibiotics. In this review, we have shown how *P. aeruginosa* and UPEC exploit quorum sensing (QS) and biofilm formation to regulate virulence, persist within host environments, and tolerate antimicrobial treatments. Their signaling networks, such as the Las/Rhl/PQS/IQS hierarchy in *P. aeruginosa* and the LuxS/AI-2, AI-3/QseC, and indole systems in UPEC, govern key pathogenic traits, including biofilm development, metabolic adaptation, efflux activity, and the emergence of persister cells, all of which contribute to recurrent and difficult-to-treat infections.

Recent advances in computational biology, structural modeling, and proteomics are transforming the way these regulatory systems can be analyzed and targeted. Deep-learning tools now provide high-quality structural predictions for QS-related proteins lacking experimental characterization, enabling structure-based virtual screening, pharmacophore modeling, and molecular dynamics simulations. When integrated with ligand-based methods and machine-learning QSAR approaches, these tools support the rapid identification and prioritization of compounds, particularly natural products, with potential anti-QS or anti-biofilm activity.

Among these compounds, plant-derived metabolites have emerged as a promising but still underexplored source of QSIs. Phytochemicals such as flavonoids, alkaloids, terpenoids, and phenolic derivatives exhibit multi-target activity and can attenuate QS-regulated functions without exerting strong selective pressure. Coupling natural product databases within in silico screening pipelines is accelerating the discovery of molecules capable of disrupting autoinducer synthesis, receptor activation, or downstream transcriptional regulation.

## 9. Future Perspectives

Looking ahead, future research should increasingly focus on integrated bioinformatics workflows that combine deep learning–based screening approaches with molecular docking, molecular dynamics simulations, and high-resolution experimental validation, including proteomic and systems-level analyses. Such integrated pipelines could improve hit prioritization and help bridge the gap between in silico predictions and biological relevance. Expanding the explored chemical space, particularly by targeting underrepresented or poorly characterized classes of plant metabolites, may reveal novel scaffolds with enhanced potency, improved selectivity, or multi-pathway activity. Another promising direction lies in the development of inhibitors capable of targeting multiple nodes within QS networks, such as simultaneously modulating Las, Rhl, and PQS systems in *P. aeruginosa* or LuxS- and QseC-mediated signaling in UPEC, thereby reducing compensatory responses and increasing robustness of QS disruption.

From an experimental standpoint, the establishment of more standardized and physiologically relevant in vitro and in vivo models remains a priority, as current biofilm assays often fail to fully capture the complexity of clinical environments and polymicrobial infections. Finally, combination therapies pairing QS inhibitors with conventional antibiotics warrant further investigation, as such strategies may enhance treatment efficacy, lower effective antibiotic doses, and limit the emergence of resistance.

Altogether, the convergence of advanced computational methods, natural product discovery, and refined experimental validation holds significant promise for accelerating the development of sustainable anti-virulence therapies against multidrug-resistant pathogens.

## Figures and Tables

**Table 1:** Representative selection of widely used free and open-source tools for virtual screening, molecular docking, and molecular dynamics simulations in drug discovery workflows.

Category	Tools	Description
Natural Compounds Databases	COCONUT—Collection of Open Natural Products [189], SuperNatural 3.0 [190], NPASS—Natural Product Activity and Species Source [209], CMNPD—Comprehensive Marine Natural Products Database [210]; Latin American Natural Product Database LANaPDB) [211], The Natural Products Atlas 3.0 [212]	Collection of Natural Products and Databases
Protein Structure Prediction—Homology-based	SwissModel [167], Phyre [168], MODELLER [169]	Template-based structure prediction
Protein Structure Prediction—Ab-initio	AlphaFold 2 [170], RoseTTAFold [171], OmegaFold [213], ESMFold [214], EquiFold [215]	Deep learning-based structure prediction
Protein Complexes Prediction	AlphaFold 3 [174], RosettaFold-All-Atoms [175], Boltz-1 [216], ColabFold [217]	Multi-protein complex modeling
Virtual Screening— Pharmacophore Modelling	Pharmit [218], ZINCPharmer [219], PHASE [220], LigandScout [221]	Pharmacophore-based screening
Virtual Screening—Molecular Docking	AutoDock4 [222], AutoDock Vina [223], Smina [224], SwissDock [225], PLANTS [226], rDock [227], Gnina [228], GOLD [229], Molegro [230], Glide [231], FlexX [232].	Docking-Based Screening
Molecular Dynamics	GROMACS [196], OpenMM [233], NAMD [234], CHARMM-GUI [235], AmberTools [236]	MD simulation software

**Table 2:** Representative QS targets in *P. aeruginosa* and UPEC, associated classes of plant-derived natural compounds, and the computational and experimental approaches reported in the literature for their investigation.

Pathogen	QS System/Target	Representative Natural Compounds	Computational Approaches	Experimental Validation	Ref
*P. aeruginosa*	LasR (3OC12-HSL receptor)	Flavonoids (e.g., Baicalein, Quercetin, Naringenin, Rutin, Catechin)	Molecular docking, molecular dynamics simulations, virtual screening	QS reporter assays; elastase and pyocyanin quantification; biofilm inhibition (crystal violet); CLSM	[182,185,186,187,259,264,265]
*P. aeruginosa*	RhlR (C4-HSL receptor)	Terpenoids, cinnamic acid derivatives	Structure-based virtual screening; molecular docking; binding energy calculations	Rhamnolipid production; swarming motility; biofilm formation assays	[184,266,267]
*P. aeruginosa*	PqsR (MvfR)/ PQS pathway	Alkyl quinones; plant-derived phenolic compounds	Structure-based docking, QSAR modeling	virulence factor assays; biofilm analysis	[184]
*P. aeruginosa*	LasI/RhlI (autoinducer synthases)	Fatty acid derivatives; plant secondary metabolites	Molecular docking	Autoinducer quantification; QS gene expression analysis	[262,267,268]
UPEC	LuxS/AI-2 system	Coumarins, flavonoids, cinnamic acid derivatives	Molecular docking	AI-2 bioluminescence assays; biofilm formation assays	[252,253,259]
UPEC	QseC (sensor kinase)	Indole derivatives, phenolic compounds	Molecular docking; network-based analysis	Motility assays; QS-regulated gene expression analysis	[249,269]
UPEC	Indole signaling (TnaA)	Indole analogues, plant-derived aromatics	Molecular docking; molecular interaction analysis	Biofilm assays; antibiotic tolerance tests	[269,270,271]

## Data Availability

No new data were created or analyzed in this study. Data sharing is not applicable to this article.
